# Pollution-Induced Allergy Skews Metabolism Toward Obesity—A Conceptual Review

**DOI:** 10.3390/cimb48020198

**Published:** 2026-02-11

**Authors:** Kaustubh Jumle, Fehmi Boufahja, Anis Ahmad Chaudhary, Manali Datta

**Affiliations:** 1 Amity Institute of Biotechnology, Amity University Rajasthan, Jaipur 303002, Rajasthan, India; 2 Department of Biology, College of Science, Imam Mohammad Ibn Saud Islamic University (IMSIU), Riyadh 11623, Saudi Arabia

**Keywords:** environmental pollutants, allergy, obesity, WAT and BAT, TRP channels, spices

## Abstract

Rapid urbanization and significant lifestyle changes have become the risk drivers for the epidemiology of diseases. With urban transitions, substantial persistence of pollutants in the environment has been observed. Epidemiological studies indicate a strong relationship between air pollution and exacerbation of asthma and other allergic diseases due to particulate matter (PM). PMs in bioaerosols and aeroallergens induce the immune response, eliciting systemic inflammation. Continuous exposure to PM_2.5_ along with gases like nitrogen oxide aggravate oxidative stress and inflammatory responses. Other pollutants elevate blood glucose, inducing poor sleep patterns which in turn induce low-grade chronic inflammation. This in turn acts as a trigger for adipocyte dysfunction and reduced energy expenditure. Taken together, air pollution, allergy, and obesity constitute a jigsaw with missing pieces. Transient Receptor Protein (TRP) channels have important roles in allergic rhinitis, systemic inflammation, adipogenesis, and obesity development, underscoring a potential role as a common mechanistic link. The goal of this review is to summarize and comprehend the intricate network connecting these “modern-day hyperendemic diseases” and the plausible role played by TRP in shaping their epidemiology. Bioactive compounds in dietary spices also modulate TRP channels. Thus, spices position themselves as potential regulators at the interface of environmental sensing, inflammation, and metabolism, indicating spice-based interventions may represent an adjunct strategy to alleviate the pollution-induced allergy and obesity risk.

## 1. Introduction

Increasing urbanization has led to emissions of numerous pollutants, resulting in adverse health effects in humans. Among them, PMshave become a major cause of concern as they penetrate the respiratory system even up to the bronchioles. Factory and automobile emissions like nitrogen oxide, carbon monoxide, sulfur dioxide, volatile Organic Compounds (VOCs), dioxins, and polycyclic aromatic hydrocarbons (PAHs) constitute part of these persistent and ubiquitous pollutants. The literature demonstrates that air pollution is one of the major factors influencing allergies. Baseline household PM_2.5_ levels generally remain the same, although short-term increases are frequently observed due to cooking-related emissions and the resuspension of indoor dust particles [1]. The airborne PM_2.5_ concentration ranges from around 50 to 400 μg/m^3^ in major cities worldwide [1] but may reach up to 800 μg/m^3^ in industrially advanced cities [2,3]. Additionally, occupational exposure from agriculture and food industries adds to combustible dust, causing long-term and short-term respiratory issues including occupational asthma, dermatitis, etc. Air pollutants tend to act as adjuvants altering the immunogenicity of allergenic proteins, thus aggravating oxidative stress and skewing the immune system toward allergic reactions [4]. PM_2.5_ presents in the troposphere and absorbs and/or deflects UVB radiation, thus diminishing the intensity of incident photons. Inadequate cutaneous absorption of solar UVB results in vitamin D deficiency; low serum vitamin D levels have been consistently associated with an increased predisposition to allergic sensitization [5]. Suspended PM initiates pulmonary irritation by pro-inflammatory responses rather than mass concentration of air pollutants [2].

Obesity is currently the fifth leading risk for global deaths with almost 2.8 million deaths per year. Persistent increased levels of PM_2.5_ and O_3_ have been found to be associated with a higher level of body mass index (BMI);in addition increased NO_2_ and SO_2_ propels a higher than optimal BMI. The global prevalence of excess body weight (BMI ≥ 25 kg/m^2^) has increased by 40% and the number of obese adults (BMI ≥ 30 kg/m^2^) has increased more than six-fold to 671 million over a period of four decades [6,7]. Obesity predisposes an individual to a pro-inflammatory state via enhanced production of IL-6 and TNF-α and reduced levels of adiponectin. IL-6 induces the production and secretion of CRP, resulting in systemic inflammation. This triggers vascular and endothelial dysfunction due to decreased nitric oxide and elevated reactive oxygen species (ROS), leading to oxidative stress. Continuous exposure to air pollutants leads to abnormalities in triglyceride lipoproteins and inflammation [8,9], resulting in dendritic cell maturation and skewing immune responses toward allergy. Children with asthma display lower physical activity levels and disturbed sleep patterns, adding asthma to the risk factors for obesity. In addition, medications prescribed for asthma, especially steroids, have also been linked to obesity [10].

Although air pollution, allergic disease, and obesity have been extensively studied in isolation, there remains a critical lack of integrative mechanistic frameworks explaining how these conditions converge at the molecular level. Current studies also leave a gap in answering whether there may be a mediator linking PM-induced hypersensitivity with inflammation and obesity. To address this, a comprehensive literature search was conducted using PubMed, Scopus, Web of Science, and Google Scholar using the keywords human epidemiological studies linking air pollution and allergy, allergy and obesity, and pollution and obesity. Articles published primarily between 2005 and 2024 were considered to capture the basics. This provided mechanistic outcomes leading to obesity- and inflammation-induced functional reprogramming of different types of adipose tissues. Studies where environmental pollutants showed correlation with allergic immune responses (asthma and allergic rhinitis) or pollution exposure showed correlation with adiposity, dyslipidemia, and adipose tissue inflammation were selected for review. Research withpollution exposure or obesity without assessment of allergic or immune components, not related to the respiratory system, or had occupational or acute toxic exposure that was not representative of chronic environmental exposure were not considered. These results helped in generating hypothesis-driven interpretation.

In this review, we aim to interpret existing evidence and to propose an emerging yet underexplored framework linking pollution-induced allergy and obesity through shared molecular mediators.

## 2. Molecular Connections of Air Pollution and Allergy

Particulates with an aerodynamic diameter of about 2.5 µm are known as suspended PM_2.5_. PM may penetrate through the epithelial layer, whereby the surface concentration of PM_2.5_ may persist for almost seven days. Upon dermal absorption, particulates interact with the plasma membrane of cells and cellular uptake may channel them to cellular organelles [11,12,13]. Their size enables easy penetration into the respiratory tract, especially airway epithelial cells (AECs), aggravating allergic rhinitis (AR) and nasal mucosa inflammation. PM promotes sensitization of the respiratory system by modulating the allergenicity of airborne allergens. Sensitization results in an increased concentration of interleukin (IL)-8, leading to IL-8-mediated inflammation in bronchoalveolar spaces, which results in augmented neutrophil-dependent airway inflammation. Systemic mediators like TNF-α tend to translocate from the respiratory network into the circulation, eliciting production of acute-phase proteins (APPs) by the liver associated with fever, anorexia, and catabolism of muscle cells [14,15].

Mediators elicited by airway epithelial cells in response to PM_2.5_ exposure, like T cells, eosinophils, and the associated cytokines, result in reduction in inhalational tolerance and airway remodeling, also known as bronchial remodeling (BR), whereby epithelial damage and fibrosis occurs followed by smooth muscle hypertrophy and hyperplasia, creating a fertile environment for triggering bronchial hyperresponsiveness (BHR). BR and BHR are interrelated and involve heightened oxidative stress followed by activation of the NF-κB and MAPK signaling pathways, which are accompanied by reduced airway barrier function and asthma [16].

In PM-induced rhinitis, suspended particles engage toll-like receptor-4, TLR4, a recognition receptor of the host immune system actively involved in host defense, inflammation, and immune regulation on nasal epithelial and resident immune cells, activating innate immune signaling cascades and inducing pro-inflammatory cytokine production via IL-6-dependent Signal Transducer and Activator of Transcription 3 (STAT3) activation. STAT3 is a transcription factor which induces the production of cytokines, thus driving transcriptional programs that amplify mucosal inflammation, compromise epithelial barrier integrity, enhance immune cell recruitment, and sustain chronic rhinitis pathology [17].

One class of cellular membrane receptor that facilitates binding of PM_2.5_ is the TRP proteins. Different types of TRP receptors are activated by different stimuli like airway exposure, acute respiratory events, and chronic inflammatory disease of the respiratory system. The subtypes, TRP vanilloid 1 (TRPV1) and ankyrin 1 (TRPA1), are found in nociceptive C fibers of the AECs and their smooth muscle (ASM) cells. These chemoreceptors are activated by environmental particulates like diesel exhaust particles (DEPs), ozone, cigarette smoke, and PMs. TRPV1 and TRPA1 channels may also be activated by oxidative stress and ROS such as superoxide and hydrogen peroxide [1].

Activation of TRPV1 triggers Ca^2+^ influx-induced activated mast cells, which in turn results in the release of pro-inflammatory elicitors like neurokinin A (NKA), tachykinin substance P, and calcitonin gene-related peptide (CGRP), along with leukotrienes, TNF-α, and IL-1β. These elicitors in turn induce neurogenic inflammation by stimulating the vagus nerve, olfactory nerve, nasopharyngeal nerve, and trigeminal nerve, thus modulating the cough threshold [18].

PMs aggravate TRP expression via BR and BHR; these pathways induce expression of epithelial alarmins like IL-33, TSLP, and IL-25, which in turn enhances TRP receptor expression and thus activity [19,20]. This creates a feed-forward loop, amplifying allergic inflammation in the nasal mucosa.

## 3. Molecular Connections of Allergy and Obesity

Allergic rhinitis (AR) is an IgE-mediated inflammatory disease induced by allergen exposure demonstrating symptoms like rhinorrhea, itching, sneezing, bronchial hyperresponsiveness, and airflow obstruction. It has been indicated that allergies and obesity form part of a vicious cycle, where each of them induces the other, inadvertently increasing the propensity to develop airway inflammation. There is a strikingly high prevalence of obesity among individuals with asthma. Although about one-third of the U.S. population is obese, many recent studies of asthmatic populations report a prevalence of obesity of 50% or more in these individuals [21,22].

Obesity and allergic disease, which are health problems for both developed and developing countries, are increasingly being seen in childhood worldwide. The relationship between obesity and asthma is complex and multifactorial. Many reasonable mechanisms have been suggested, including a shared genetic component, dietary and nutritional factors, changes in the gut microbiome, systemic inflammation, metabolic abnormalities, and changes in lung anatomy and function [3,23].

**Figure 1:**
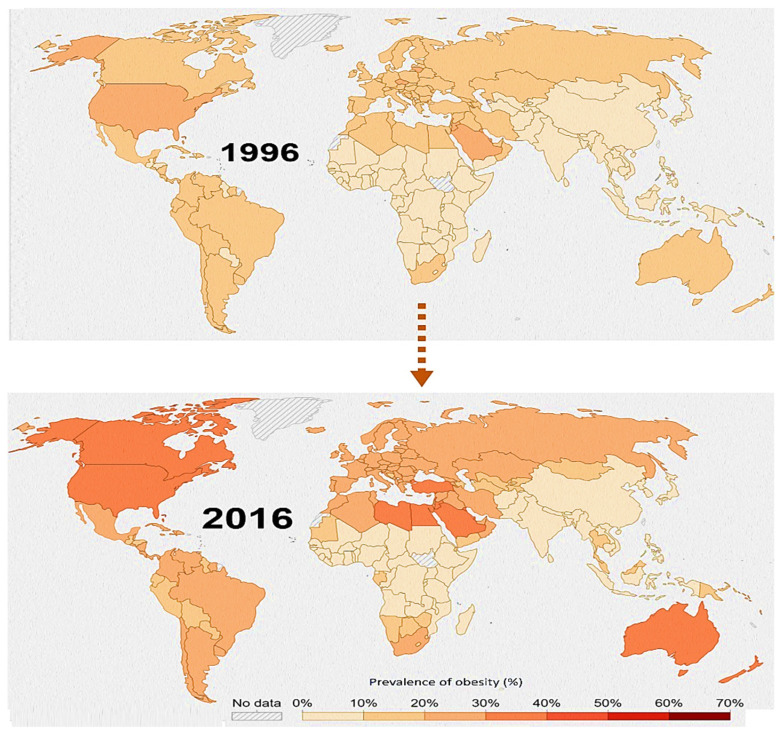
Temporal change in the global burden of adult obesity alongside monitored ambient PM_2.5_ exposure from 1996 to 2016. The scale correlates the intensity in color with the increase in prevalence of obesity as a percentage. The visualization highlights the marked rise in adult obesity prevalence over two decades and its overlap with regions experiencing elevated PM_2.5_ levels (exceeding 5 µg/m^3^) [Source https://www.healthdata.org/data-tools-practices/interactive-visuals/gbd-results, accessed on 22 September 2025].

Children with allergies have a tendency to develop obesity, high blood pressure, and high cholesterol. Over a decade, the prevalence of the obese population has increased many-fold [Figure 1] and so have allergic manifestations like asthma rhinitis and atopic dermatitis (WHO https://www.who.int/data (accessed on 15 November 2025)). The pathogenesis of asthma in obesity using animal models indicates that obesity may induce an asthma-like phenotype through innate, non-Th2 pathways. A human homolog of Chi3l1 (YKL-40) increases in the serum of asthmatic patients and is associated with truncal obesity, thus providing an initial corroboration that allergy and obese asthma might be intricately related [24,25,26,27,28].

Adipose tissue has been categorized into two major types: brown (BAT) and white adipose tissue (WAT). BAT mainly regulates energy expenditure by thermogenesis and is inversely correlated with body mass index (BMI). WAT regulates immunity and inflammation as macrophages, especially CD14^+^ and CD31^+^, constitute 10% of its stromal vascular fraction and enable secretion of cytokines and chemokines from eosinophils [29], such as tumor necrosis factor α (TNFα), interleukin 6 (IL-6), interleukin 10 (IL-10), interleukin 1β (IL-1β), and other factors, such as monocyte chemoattractant protein-1 (MCP-1). TNFα induces secretion of IL4 and IL5, skewing the immune system towards a Th2 cytokine profile, thus increasing the risk of allergy [30,31,32]. Another class of brown-like adipocyte aptly termed beige/brite adipocyte has been categorized, which forms an inducible form of human WAT. WAT upon stimulation converts itself into beige adipocytes mimicking the properties of BAT [33].

Whether localized inflammation contributes to increased WAT accumulation remains unclear. Do allergens play any role in activation of the adipocytes? And do TRPs play any role in this?

Exposure to allergens aggravates an inflammatory response by recruiting neutrophils to the airways. Allergens like ragweed pollen stimulate CXCL-8 secretion from cells expressing toll-like receptor-4 (TLR-4), and Myeloid differentiation protein-2 (MD-2) attracts ROS-generating neutrophils [34]. Another well-known allergen, Der p 2, facilitates the TLR4 inflammatory cascade. In the early stages of obesity, neutrophils infiltrate into adipose tissue, where they produce chemokines and cytokines, thereby promoting macrophage infiltration [35,36]. Higher levels of NF-κB in the vicinity of adipocytes result in elevated secretion of IL-1β by WAT-infiltrated neutrophils. Epithelial alarmins like IL-33 released due to BR and BHR activate the type 2 innate lymphoid cells (ILC2s) in WAT. Upon induction ILC2s produce methionine-enkephalin peptides and IL-13, which induce UCP1 expression in adipocytes, promoting BAT-like activity [35]. Thus, allergic sensitization involves crosstalk between TLR4-induced mucosal inflammation and MD-2-dependent signaling, resulting in elevated ROS. The continual presence of ROS disrupts oxidative damage to cellular components. It also impairs mitochondrial function and fatty acid oxidation, causing lipids to accumulate in tissues. Perturbed lipid metabolism promotes enhanced lipolysis and a consequent increase in free fatty acid (FFA) availability [36].

The presence of FFA results in activation of 5-lox-expressing cells in the WAT and the production of LTB4, thereby inducing further neutrophil accumulation in the WAT. FFAs additionally activate inflammasomes in WAT neutrophils. Persistent presence of FFAs results in an expansion in the number and size of adipocytes; the number keeps on increasing till limited anabolic capability due to expansion limitations. This is further ensued by an inflammatory pathway induction in response to this stress [12,36]. With an increase in body weight, circulating levels of IL-6, leptin, and TNF-α increases, which in turn leads to suppression of the activity of regulatory T lymphocytes (Tregs) [37].

A decrease in adiponectin downregulates the secretion of IL10, resulting in decreased immunological tolerance to antigens. Thus, the correlation of obesity and allergy forms an intricate epidemiological network culminating in a vicious cycle [38]. TRPs are expressed on both WAT and BAT, but the TRPs in the adipose tissues are more involved in the thermogenesis of the adipocytes. TRPV1 expression levels have been found to be higher in brown adipocytes than in undifferentiated pre-adipocytes. TRPs facilitate entry and modulation of intracellular Ca^2+^ concentration, which in turn regulates adipocyte behavior depending on their differentiation stage. Similarly, other members of the TRPV family have been observed to induce thermogenesis via the PGC1α/UCP1 pathway. However, other members of the TRP channels modulate intracellular Ca^2+^ dynamics and exert biphasic control on differentiated adipocytes [39,40].

## 4. Pollutants Exacerbate Obesity—An Indicative Investigation

**Figure 2:**
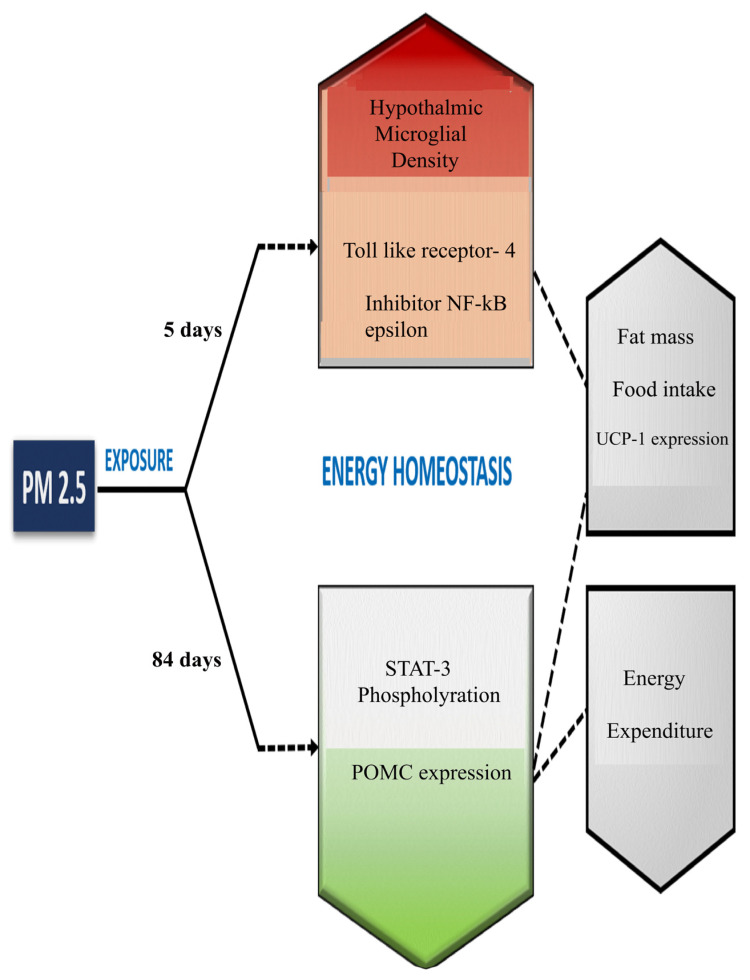
Systemic effect of short-term [5 days] and long-term [84 days] exposure to PM_2.5_ matter on energy homeostasis. Short-term PM_2.5_ exposure triggers acute hypothalamic neuroinflammation marked by microglial activation and TLR-4–NF-κB signaling, leading to early alterations in food intake, fat mass, and thermogenic responses, whereas prolonged exposure causes chronic disruption of hypothalamic STAT3–POMC signaling, resulting in impaired energy expenditure and sustained dysregulation of energy homeostasis [42,43,44].

With many common mediators amongst the pathway aggravating pollution-based allergies and allergy-based obesity, a possibility arises that pollutants may induce obesity in individuals. A cross-sectional study conducted in China established a relationship between air pollution and obesity, whereby a 10 μg/m^3^ increase in PM_2.5_ exposure may increase obesity risk by 8% [41]. Data indicates the global burden of PM_2.5_ is exceeding the permissible limits in most of the continents [Figure 1]. There has been a marked 30% increase in childhood obesity in relation to air pollution [42,43]. Exposure to PM_2.5_ increases the expression ofTLR-4, which in turn recruits adapter protein 88 (MyD88) and Tank 1 protein kinase to induce inflammatory pathways as well as innate and adaptive immune responses. Leptin-mediated STAT3 phosphorylation is essential for the expression of proopiomelanocortin (POMC), the physiological regulator maintaining the fine balance between energy balance and body weight. PM_2.5_ exposure of >12 weeks results in dysregulation in the phosphorylation of STAT3 and thus leptin resistance [44,45,46,47,48] [Figure 2].

The mixture of traffic-related air pollutants (TRAPs) and criterion pollutants constitute the eco-exposome persisting in a particular site [45]. In the adult population, continuous exposure to PM_10_ exerts the greatest effects on obesity. Although the spatial, temporal, and intra-individual variation is apparent, the correlation between exposome and obesity has been widely neglected. The HELIX (Human Early-Life Exposome) study indicated that the presence of indoor smoke acts as an obesogen, resulting in higher body mass indexes [40]. Criterion pollutants like dichlorodiphenyltrichloroethane (DDT) and its metabolite dichlorodiphenylethylene (DDE) are endocrine disruptors (EDCs) and proven to be associated with increased obesity risk. Ambient PMs induce insulin resistance associated with BAT mitochondrial dysfunction. On the other hand, prolonged exposure to TRAP results in heightened lipolysis, which in turn results in accumulation of acylcarnitines (ACTs) [45,49].

ACTs are fatty acid bound carnitine moieties present on the outer mitochondria membrane and are broken down by beta-oxidation for energy production. Long-chain ACTs play a role in insulin resistance and the development of cardiovascular diseases. Accumulation of ACTs and ROS contributes to metabolic stress and impairs insulin signaling, eventually resulting in lipogenesis. ACTs evade mitochondrial entry and are exported to the blood plasma and C3–C6 ACTs have been found to be significantly persistent in obese individuals. ACTs have been known to induce the secretion of inflammatory cytokines through activation of the toll-like receptor/MyD88 signaling-mediated NF-κB pathway [43,50,51]. In addition to mimicking obesity-like plasma concentrations of ACTs, the allergens tend to induce obesity via the respiratory pathway by aggravating the inflammatory pathways; inflammation begins in the fat cells mainly due to dysfunction of the mitochondria. Infusion of inflammatory cytokines results in insulin resistance, which is symptomatic of type 2 diabetes mellitus. Chronic cases of inflammation eventually result in leptin resistance which in turn impairs glucose and fat metabolism, resulting in weight gain and insulin resistance [48].

Epidemiological evidence from 2015 to 2024 consistently associates PM_2.5_ and traffic-related pollutants with both allergic disease and obesity-related outcomes in adults, although these relationships remain associative and context-dependent [Table 1].

## 5. The Connecting Link—TRP

**Figure 3:**
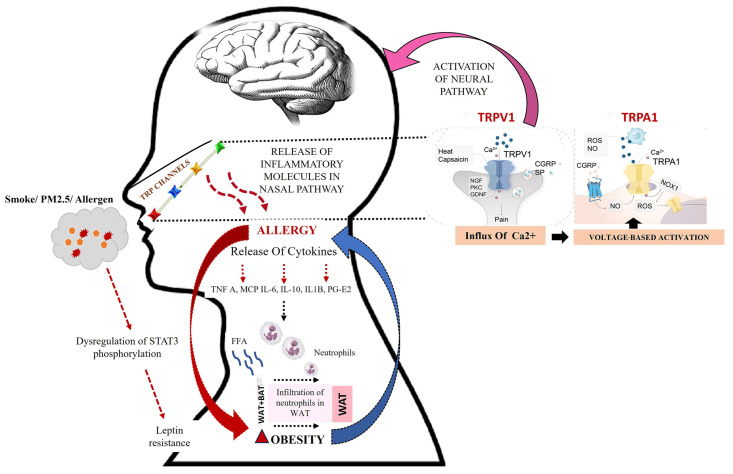
Schematic of PM affecting the propensity to obesity. Inhaled environmental pollutants and allergens activate TRPV1 and TRPA1, inducing Ca^2+^ influx and neuropeptide (CGRP, Substance P) release, leading to neurogenic inflammation and allergic responses. Peripheral inflammation communicates with the central nervous system, activating hypothalamic microglia and disrupting NF-κB–STAT3 signaling. This results in leptin resistance and altered energy homeostasis. Downstream, inflammatory cytokines (TNF-α, MCP-1, IL-6, IL-1β, and PGE2) promote neutrophil infiltration and WAT expansion (solid red arrow), driving obesity. In parallel, TRP-mediated cytokine reprogramming can induce UCP1 expression and facilitate WAT-to-BAT transition (dashed black arrows), enhancing thermogenesis and energy expenditure. (Red arrow indicates obesity induction; blue arrow: obesity-aggravating allergy; pink arrow: neural activation of allergy) [37,39,49,50,51,53,58,59,60,61,62,63].

As these risk factors do not exist in isolation, populations tend to experience simultaneous manifestations of the result. One of the common factors which is evident in all interactions is TRP. TRP constitutes a family of cation channels that may be categorized in seven subfamilies based on their amino acid sequence similarities. These are canonical (TRPC), vanilloid (TRPV), melastatin (TRPM), TRPA (Ankyrin 1), polycystic (TRPP), NOPMC-like (TRPN), and mucolopin (TRPML) [Figure 3]. Activation of TRP channels leads to an influx of mainly Ca^2+^ and Na^+^, which in turn regulate multiple intracellular biochemical signaling pathways. Predominantly present in the upper airway, they form the key mediators for chemosensation, thermosensation, nociception, release of neuropeptides and immune cell mediators, and mucus secretion. Nanoparticles form an active part of the PM_2.5_ fraction and they activate the TRPs by mechanical perturbation [Figure 3]. Gaseous pollutants tend to chemically modify and thus activate the TRP channels. Coal fly ash, another common ingredient of PM_2.5_, results in pro-inflammatory and pro-apoptotic responses by activating TRPM8, TRPV1, and TRPA1 [14,56,57].

TRPA1 augments vagal nerve discharges to induce pain, cough, and inflammation. This channel may be modulated with Ca^2+^, trace metals, pH, ROS, nitrogen, and carbonyl species. TRPV1 is activated by abnormal temperature, acidic pH, and lipid derivatives and is sensitive to vanilloid molecules, like capsaicin. In the nasal epithelium, TRPV1 and TRPA1 activity is modulated in an intense crossplay of phosphatidylinositol-4,5-biphosphate (PIP_2_) and prostaglandin E2 [Figure 3] [64,65].

Patients with asthma and continuous exposure to allergens become more sensitive to TRPV1 because of the acidic environment of the airways and thus to BR and BHR. Once the PM_2.5_ reaches the lower respiratory tract, it tends to deposit in the alveoli and exacerbate inflammation via the TRM8, TRPV1, and TRPA1 channels, wherebyTRM8 is most prone to activation by PMs. TRPV1 and TRPM2 increase the gaps in the peripheral membranes of the tissues due to movement of Zona occludens-1 (ZO-1) proteins, resulting in vascular hyperpermeability. An increase in permeability results in aggravation of PM-induced allergy and asthma [49,66].

Upregulated expression of TRPA1 and TRPV1 due to alarmins increases susceptibility to cough reflexes as an after-effect of sensitization by bradykinin and NGF. The coughing reflex in human results in augmented expression of TRPV1 on sensory nerves [61,62], thus forming a vicious cycle.

Activation of TRPV1 in the nerves of CNS and PNS release neuropeptides, inducing heightened coughing reflexes due to the contraction of smooth muscles. Consistent coughing will result in systemic inflammation and thus mechanical stretch and hydropic degeneration, resulting in activation of TRPV2. Activated TRPV2 in turn demonstrates heavier WAT and increased lipid accumulation in BAT. Moreover, with a high-fat diet, there is a significant increase in body weight and fat in metabolically active tissues [67,68,69].

TRPM8 expressed in WAT has heightened expression levels during the differentiation of adipocyte. TRPM8 activation induces UCP1 expression, mitochondrial activation, and heat production. Activated TRP channels (TRPV1, TRPV4, TRPM3, TRPM8, and TRPA1) enable crosstalk between neurons, immune cells, and epithelial cells to regulate a wide range of inflammatory actions [70] via the MAPK signaling pathway. As already mentioned, TRPV1 is present in both WAT and BAT; concentration being higher in BAT rather than in pre-adipocytes. Dietary TRPV1 activation in the adipocytes and WAT induces thermogenic gene expression, skewing the balance towards the formation of BAT from adipocytes. Previous studies indicated that TRPV1-induced activation of TRPV4 suppresses thermogenic gene expression in adipocytes, thus downregulating thermogenesis. Upregulated expression of UCP1 suppresses accumulation of WAT in spite of a high-fat diet in mice [51,62].

A study by [71] indicated that expression of TRPV4 suppresses thermogenic genes and modulates mitochondria, thus suppressing the browning of WAT. Pharmacological inhibition of TRPV4 tends to enable lower inflammation, increases adipogenic and lipogenic gene expression, and leads to larger lipid droplets. Thus, TRPV4 may act as a therapeutic target for obesity and metabolic syndrome [71].

Insights into the mechanistic role of TRPs indicate that these polymodal sensors have unique and sensitive responses to different stimuli. Even the site of stimulus may decide the fate of the response. Activation of the TRP channel via the respiratory pathway triggers increased expression of pro-inflammatory markers, which in turn results in obesity. In contrast, direct activation of TRPs present on WAT and adipocytes can convert “bad” fat into “good” fat. Can this critical information help in designing interventional therapy to curb pollution- and allergy-induced obesity?

## 6. Spices as Modulators of TRP

Spice has been termed as a Darwinian gastronomic ingredient, whereby it has been used for its co-evolutionary role [72]. With epidemiological studies, spices have been recognized for their potential health benefits, including their role in alleviating certain diseases [Table 2] [44,73]. In recent times, consumption of spices has shown a correlation with fewer COVID-19 cases and higher recovery rates. With spices having a multifarious role in various diseases, one of the main targets of the phytochemicals from spices seems to be TRPs. This raises the possibility that ingredients from spices may play a role in regulating TRP-based modulation [74].

Active natural compounds have contributed as holistic alternatives for improving human well-being. Natural products have been actively used to alleviate symptoms for various human conditions, such as chronic inflammatory diseases, metabolic disorders including obesity, cardiovascular ailments, respiratory infections, and gastrointestinal disturbances. Is there a possibility that nature has an alternative present for the slow-acting modern epidemic? Several of the same ion channels implicated in inflammation-driven obesity also interact with bioactive metabolites derived from dietary spices [Table 3].

The vanilloids consisting of molecules like vanillin, resiniferatoxin (RTX), acetovanillone, vanillyl mandelic acid, homovanillic acid, and capsaicin, capable of regulating neurogenic inflammation and a variety of physiological reflexes and local regulatory functions. As vanilloids have an inherent characteristic to bind to the TRPV channels, desensitization to vanilloids is considered one of therapeutic approaches to subdue neuropathic pain and symptoms of vasomotor rhinitis [77,78]. PM-driven TRPV activation induces neurogenic inflammation, intricately connecting inflammation, sleep disorders, [79] and obesity; vanilloids may enable diminishing it. Studies indicate vanilloids enhanced BAT thermogenesis with a subsequent decrease in fat mass in humans, thus reinstating that TRPV may be the mediator [61].

**Table 3:** Different classes of TRP channels with their agonists and their different activation mechanisms [79,80,81,82,83,84].

Channel	Activated in	Agonists from Spices (Dietary)	
TRPV1	Neuropathic pain Visceral pain Inflammatory pain Itch	Capsaicin Allicin 4-hydroxynonenal	Piperine Eugenol Vanillin Gingerol
TRPV4	Mechanical pain Neuropathic pain Visceral pain Trigeminal pain Inflammatory pain	Endothelin Histamine Serotonin	
TRPA1	Nociceptive pain Inflammatory pain Hereditary episodic pain	Mustard oil Cinnamaldehyde Allicin	4-hydroxynonenal Carvacrol
TRPM8	Cold hypersensitivity Neuropathic pain Orofacial pain	Menthol Geraniol Icillin	Eugenol Poly Unsaturated Fatty Acid Lyso-phosphatidylcholine

Capsaicin ingestion enhances fat oxidation and energy metabolism; upon intake of capsaicin analogs for a period of 1–3 months, increased fat oxidation with a reduction in abdominal fat deposits was detected in humans. Capsaicin binds to TRPV1 in the GI tract, resulting in elicitation of the hypothalamic nucleus of the central nervous system, which is then transduced to WAT. This further induces expression of PRDM16 protein, promoting the formation of beige adipocytes and thus increasing systemic energy expenditure [61]. TRPV1 is sensitive to capsaicin concentrations ranging between 0.1 and 10 µM. But, continuous exposure to capsaicin leads to desensitization of TRPV1 and thus dysregulation, leading to obesity [63,73].

Essential oils consisting of monoterpenes, aromatics, and sesquiterpenes possess a broad-spectrum therapeutic potential against obesity and its related diseases. EOs like eugenol and 1,8-cineole stimulate TRP receptors, increasing energy expenditure and thermogenesis, reducing the appetite and release of ghrelin. These compounds increase mitochondrial biogenesis in WAT and activate BAT. Trans-anethole (TA), an active flavoring agent present in EOs, reduces adipogenesis and lipogenesis and increases lipolysis and fat oxidation. TA is a potent agonist of TRPA1, mediating the lipid metabolism via SIRT1 [67,74].

While PM_2.5_-based activation contributes to metabolic dysregulation and adiposity, dietary or transient TRPA1 activation enhances energy expenditure and limits adiposity. Dietary activation of TRPA1 increases GLP-1 secretion, improves glucose tolerance, and modulates gut–brain signaling relevant to energy balance [71]. Isothiocyanates and thiosulfinates from wasabi, mustard horseradish, garlic, and onion and unsaturated aldehydes like cinnamaldehyde (cinnamon), cuminaldehyde (cumin), p-anisaldehyde (anise), and tiglic aldehyde (onion/garlic) activates TRPA1 specifically. Catechins in green tea have also been observed to activate TRPV1/TRPA1 via gastrointestinal sensory functions and trigger BAT formation [75,76,77].

Data reveals consistent but controlled consumption of spices is associated with a lower prevalence of obesity in humans. In Asian countries, there is a consistent intake of spices and it is highly likely that TRPA1/TRPV1 receptors are permanently desensitized, enabling the population to take large amounts without side effects. This has resulted in enhanced energy metabolism with comparatively less prevalence of human obesity in eastern Asian countries [9,14,78]. It is thus envisioned that TRP channels have themselves revealed a way to circumvent obesity caused by environmental pollutants and allergy.

## 7. Conclusions

We hypothesize that pollutants, especially PM_2.5_, can aggravate symptomatic allergic reactions, which in turn have the capability of inducing obesity from molecular levels. Conversely, an obese person is prone to heightened allergic responses to PM_2.5._ This makes obesity and allergy part of the vicious cycle induced and piqued by PM_2.5_. Current interventions treat downstream consequences—antihistamines for allergy, steroids for inflammation, or drugs targeting obesity and insulin resistance. In contrast, PM_2.5_ is sensed by TRP channels expressed on airway epithelium, sensory neurons, and immune cells; hence, these channels may become one of the mechanisms whereby metabolic reprogramming for WAT occurs.

Despite growing interest in TRP channels as integrators of allergic inflammation and metabolic dysfunction, several limitations and knowledge gaps remain. Direct causal evidence linking allergy-induced TRP activation to obesity is limited. Furthermore, redundancy and crosstalk among TRP subtypes challenge the attribution of effects to single channels [27]. Competing hypotheses propose that obesity primarily amplifies allergic inflammation via adipokines and systemic low-grade inflammation rather than allergy driving obesity [79]. Others emphasize neuroendocrine dysregulation, microbiome alterations, or socioeconomic and behavioral determinants as dominant drivers [80].

Future studies integrating longitudinal human data, precise exposure assessment, and cell-specific TRP modulation are essential to resolve these uncertainties. Although many studies with dietary spices have been highlighted for gastrointestinal problems [81,85], cholesterol and glucose metabolism, and immune boosters [83], to date, the correlational studies of spice intake with allergy-induced obesity are lacking. Human epidemiological studies in large cohorts [85,86,87] indicate that frequent spicy food intake is associated with higher odds of being overweight and obesity compared with no spicy food consumption; however, a direct correlative link between allergy-driven inflammation and obesity in these populations remains insufficiently established [88]. Along the same lines, research indicates that the intake of spices in moderate quantities has the potential to alleviate the molecular response induced by these hyperendemic diseases. Natural spice metabolites (e.g., curcumin, capsaicin, cinnamaldehyde, gingerol, and piperine) can modulate TRP activation [Table 2] at the point of environmental sensing, thereby preventing initiation of allergic cascades that later affect WAT-BAT transitions. Dietary spices act as partial agonists or desensitizers, restoring homeostatic TRP signaling instead of abolishing it. Thus, they may offer improved safety, particularly relevant for long-term exposure to air pollution.

This opens new avenues for in-depth exploration of molecular and clinical pathways followed by randomized multicenter trials to establish the importance of spice intake with respect to allergy-induced obesity.

## Figures and Tables

**Table 1:** Epidemiological studies (2015–2024) linking pollution with allergy and obesity.

Study/Population Type	Pollutant Exposure	Allergic Outcomes	Metabolic Outcomes	Reference
Nurses’ Health Study (USA) (Adult women)	PM_2.5_ (10–30 µg/m^3^)	Adult-onset asthma	Weight gain, insulin resistance	[52]
MESA Air (USA) Multi-ethnic adults	PM_2.5_ (9.2 to 22.6 µg/m^3^), NO_2_	Asthma, airway inflammation	Visceral adiposity, metabolic syndrome	[53]
China Kadoorie Biobank Adults	PM_2.5_ (>25–50 µg/m^3^)	Respiratory/allergic symptoms	Obesity, diabetes risk	
KNHANES (Korea) Adults	PM_10_ (45–52 µg/m^3^), NO_2_	Allergic rhinitis, asthma	Obesity, dyslipidemia	[54]
Taiwan National Health Cohorts Adults + Children	PM_2.5_ (10–25 µg/m^3^)	Asthma incidence	Obesity risk	[55]

**Table 2:** Different spices with their health benefits [63,73,74,75,76,77].

Spice	Scientific Name	Active Compounds	Health Benefits									
			1	2	3	4	5	6	7	8	9	10
Turmeric	*Curcuma longa*	Curcumin										
Ginger	*Zingiber officinale*	Gingerol, Shogaol										
Garlic	*Allium sativum*	Allicin, Sulfur compounds										
Cinnamon	*Cinnamomum verum*	Cinnamaldehyde, Eugenol										
Clove	*Syzygium aromaticum*	Eugenol										
Black Pepper	*Piper nigrum*	Piperine										
Cardamom	*Elettaria cardamomum*	Cineole, Terpinene										
Cumin	*Cuminum cyminum*	Cuminaldehyde, Terpenes										
Fenugreek	*Trigonella foenum-graecum*	Saponins, fiber										
Saffron	*Crocus sativus*	Crocin, Safranal										
Bay	*Laurus nobilis*	Cineole, Eugenol										
Carom	*Trachyspermum ammi*	Thymol										
Fennel	*Foeniculum vulgare*	Anethole, Fenchone										
Asafoetida	*Ferula asafoetida*	Ferulic acid, Sulfur compounds										
Coriander	*Coriandrum sativum*	Linalool, Borneol										
Mustard	*Brassica juncea*	Glucosinolates, Isothiocyanates										
Nutmeg	*Myristica fragrans*	Myristicin, Elemicin, Macelignan										
Rosemary	*Rosmarinus officinalis*	Carnosic acid, Rosmarinic acid, Camphor, Ursolic acid										
		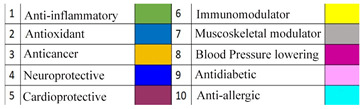										

## Data Availability

No new data were created or analyzed in this study. Data sharing is not applicable to this article.
